# TP53 Mutation Mapping in Advanced Non-Small Cell Lung Cancer: A Real-World Retrospective Cohort Study

**DOI:** 10.3390/curroncol29100582

**Published:** 2022-10-04

**Authors:** Fang Hao, Liyan Gu, Diansheng Zhong

**Affiliations:** Department of Oncology, Tianjin Medical University General Hospital, Tianjin 300052, China

**Keywords:** TP53, non-small cell lung cancer, co-mutation status, therapy response

## Abstract

Background: TP53 is frequently mutated in solid tumors, but its basic mutation mapping is mixed, particularly in aggressive-stage lung cancer. Experimental Design: We curated a total of 139 advanced non-small cell lung cancer (NSCLC) patients who harbored wild-type TP53 (TP53wt) or mutated TP53 (TP53mut) based on next-generation sequencing (NGS) to analyze multiple-dimensional data types, including tumor mutation burden (TMB), programmed death receptor ligand 1 (PD-L1) expression, co-mutant alterations, hotspot mutations distribution, and therapy response. Results: TP53 was evident in 125 mutations and significantly associated with male sex, adenocarcinoma differentiation, smoking history, PD-L1 tumor proportion score, and TMB level. The most frequent mutations were distributed on exon 8, but there were no distinct hotspot mutations. After outlining the co-mutation genes, it is interesting to note that DNA damage repair (DDR) genes were frequent alterations in the mutated TP53 cohort. Even though there was no significant difference between the TP53wt and TP53mut cohorts on therapy response, patients with nucleotide variation in G>T achieved a relatively higher durable clinical benefit (DCB) rate. Conclusions: This real-world retrospective study suggests that molecular stratification on the basis of TP53 mutations should be deeply explored for NSCLC to optimize and modify clinical therapy choices.

## 1. Introduction

Lung cancer is one of the leading causes of cancer-related mortality worldwide, with a poor 5-year survival rate of 19% [1]. There are two main forms of lung cancer: small cell lung cancer (SCLC, ~15% of patients) and non-small cell lung cancer (NSCLC, ~85% of patients), with the latter further subdivided into two main types: lung adenocarcinoma (LUAD) and lung squamous cell carcinoma (LSCC) [2]. Nowadays, an in-depth understanding of genetic alteration and molecular profiling based on next-generation sequencing (NGS) has dramatically changed the treatment landscape of lung cancer to precision therapy.

The tumor suppressor p53 (TP53) gene encodes p53 protein, which consists of 393 amino acids, with four major functional domains: transcriptional, DNA binding, tetramerization, and regulatory domains [3]. Mutations in p53 occur in approximately half of the solid tumors, resulting in loss of tumor suppressor function (impaired expression) or gain of oncogenic activity (aberrant expression). Other than DNA damage response, the exact role of p53 in tumor suppression has been attributed to intracellular metabolism, genetic and epigenetic stability, inflammation remodeling, and integration with various pathways [4,5]. It is observed that amino acid locations at 175, 245, 248, 249, 273, and 282 are frequent hotspot mutations in most cancers and mutant p53 activity may alter tumor therapy response [6,7]. Hence, the understanding of the relationship between p53 modification and tumor inflammatory environment may contribute to more effective cancer treatment. This real-world retrospective cohort study included patients with advanced NSCLC and focused on the roles of both wild-type and mutant forms of p53 in the regulation of tumor development, such as tumor mutational burden (TMB), the level of programmed death receptor ligand 1 (PD-L1) protein on the surface of tumor tissue, their interplay with other genetic alterations and therapy response.

## 2. Materials and Methods

### 2.1. Patients and Eligibility

**Figure 1:**
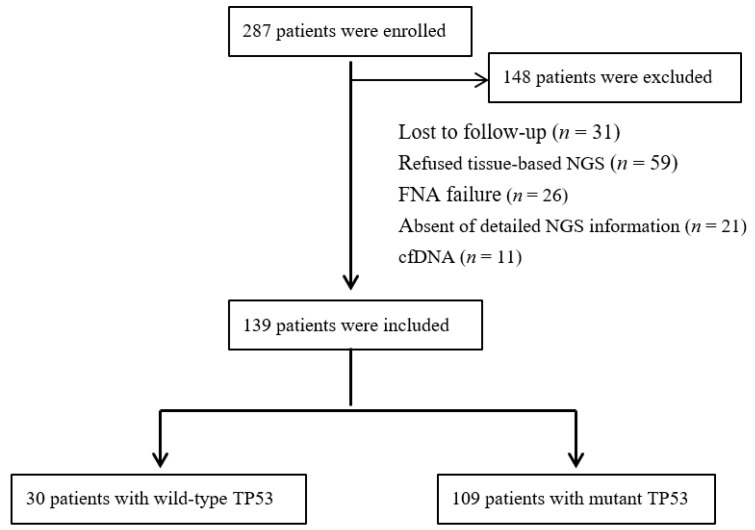
Flowchart of patient selection.

The study retrospectively reviewed 287 patients with advanced lung cancer at our Oncology Department between December 2019 and January 2022. The exclusion criteria were as follows: lost to follow-up (*n* = 31), refused tissue-based next-generation sequencing (NGS) (*n* = 59), failed fine-needle aspiration (FNA) biopsy for routinely screened of molecular alterations (*n* = 26), absence of detailed NGS information (*n* = 21), and evaluating mutational status with blood-cell-free DNA (cfDNA) (*n* = 11). Finally, a total of 109 patients with wild-type TP53 or mutant TP53 reported on NGS panels met the inclusion criteria in the present study (Figure 1). The study was reviewed and approved by the Ethics Committee of our department.

### 2.2. Patient Variables

The variables collected included patient demographics (such as age, sex, and smoking history), histological type (lung adenocarcinoma (LUAD), lung squamous cell carcinoma (LSCC), or sarcomatoid carcinoma), programmed death receptor ligand 1 (PD-L1), tumor mutational burden (TMB), and molecular alteration status. Durable clinical benefit (DCB) was defined as a partial response/stable disease that lasted more than 6 months, while no durable benefit (NDB) was defined as a stable disease that lasted less than 6 months. Patient outcomes were characterized by RECIST V.1.1 criteria in combination with clinical notes.

### 2.3. Tissue-Based Next-Generation Sequencing

DNA extracted from formalin-fixed tumor samples was analyzed with a comprehensive genomic profiling assay that targeted all exons of 825 cancer-related genes.

TMB: Panel_var_filter software (v1.0) was employed to filter variant annotation results, including specific types of variants (non-coding region variants, synonymous mutations), mutation frequency, reads support, variants exclude the coding region of panel 825 genes, and driver gene variants. Finally, the number of filtered variants was calculated to evaluate the TMB value.

PD-L1: Immunohistochemical technique was employed to calculate CPS and TPS values.

$$\mathrm{CPS}=\frac{\begin{array}{c} \mathrm{No}.\mathrm{PD}\text{-}L1\text{-}\mathrm{stained}\;\mathrm{cells}\\ \;\left(\mathrm{tumor}\;\mathrm{cells},\;\mathrm{lymphocytes},\mathrm{macrophages}\right) \end{array}}{\mathrm{Total}\;\mathrm{No}.\mathrm{of}\;\mathrm{viable}\;\mathrm{tumor}\;\mathrm{cells}}\times 100\;\mathrm{TPS}\left(\%\right)=\frac{\mathrm{No}.\mathrm{PD}\text{-}L1\text{-}\mathrm{stained}\;\mathrm{tumor}\;\mathrm{cells}}{\mathrm{Tolal}\;\mathrm{No}.\mathrm{of}\;\mathrm{viable}\;\mathrm{tumor}\;\mathrm{cells}}\times 100$$

### 2.4. TP53 Mutation Classification

Traditionally, there are two classification systems for TP53 analysis: mutational type and“Poeta rules” [7,8]. The former analyzed the “technical” type of TP53 mutation, while Poeta et al. revealed a classification of the missense mutation group into “disruptive” and “nondisruptive” mutations. Mutant p53 can be also divided into two subtypes, DNA contact and conformational (misfolded/unfolded) mutants [9]. Here, we employed a dichotomous classification as “wild-type” and “mutant” to analyze TP53 activity and further separated the missense mutation (termed “TP53 missense mutations”) from all other mutations, including deletion, synonymous, nonsense, insertion, and frame-shift mutations (termed “TP53other mutations”) based on next-generation sequencing.

### 2.5. Statistical Analysis

Statistical analyses were conducted using GraphPad Prism (version 9.4.0). The association of the qualitative variables was tested for by chi-square or Fisher’s exact test, depending on distributional assumptions. Scatter dot plots indicate median and 95% confidence interval (CI). All tests were two-sided, and *p*-values of <0.05 indicated statistical significance.

## 3. Results

### 3.1. Patient Outcomes

Table 1 summarized the demographics and characteristics of the patients with advanced lung cancer. The follow-up period ranged from 189 to 757 days, with a median follow-up time for censored patients of 414 days. A total of 109 patients (78.4%) carried TP53 mutations. Most of the patients were of the male sex (73.4%) and had adenocarcinoma (59.7%). More than half of the patients had a smoking history (72.7%). Mutations in TP53 were significantly associated with male sex, adenocarcinoma differentiation, smoking history, PD-L1 tumor proportion score, and TMB level.

### 3.2. Association with PD-L1 and TMB Expression

**Figure 2:**
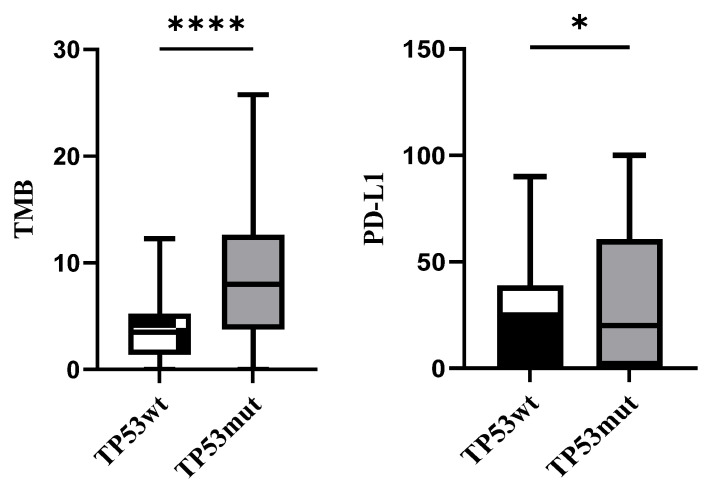
TMB and PD-L1 levels in TP53wt and TP53mut cohorts (* *p* < 0.1, **** *p* < 0.0001).

Furthermore, scatter plots for PD-L1 tumor proportion score and TMB level are shown in Figure 2. There were 30 patients with TP53wt who revealed a relatively lower expression of PD-L1 and TMB compared with 109 patients in the TP53mut cohort.

### 3.3. Mutation Mapping for TP53

**Figure 3:**
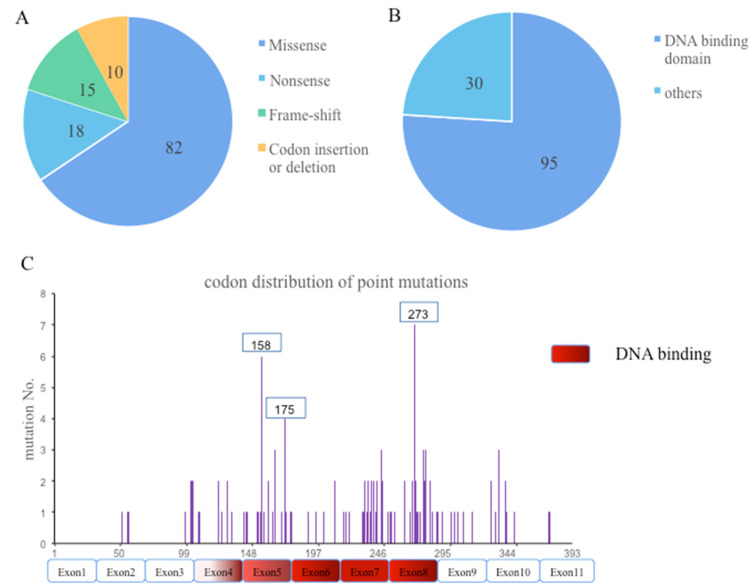
“Technical” types (**A**), cluster of domains (**B**), and codon distribution (**C**) of TP53 mutation in 109 patients.

TP53 was evident in 125 mutations, in which the majority were missense mutations (82), followed by nonsense (18), frame-shift (15), and other mutations, including insertion and deletion mutations (Figure 3A). More than half of TP53 mutations were clustered in the DNA binding domain (95), followed by tetramerization, proline-rich, regulatory, and transactivation domains (Figure 3B). The most frequent missense point mutations were distributed on exon 8(30), followed by exon 5(29), 7(28), 4(15), 9(8), 10(8), and 6(7), respectively. Accordingly, mutp53 can be divided into two subtypes, DNA contact (codons 248 and 273) and conformational (codons 157, 158, 179, 245, and 249) mutants. Here, we identified the most frequent hotspots being codons 273(7), 158(6), and 175(4). There were no distinct hotspot mutations (Figure 3C).

### 3.4. Co-Mutation Status in TP53wt and TP53mut Cohort

A series of studies demonstrate that P53 is a central tumor suppressor and the TP53 mutations display substantial immune cell composition and increased immune response [10]. Research on the co-occurring of TP53 and different mutations could help to translate the molecular findings into clinical decision-making. Here, we employed beneficial and noxious genes related to cancer treatment to evaluate their correlation with TP53wt and TP53mut. For mutated TP53, clinically relevant commutations were distributed as follows: 22.9% had EGFR mutations, 15.6% KRAS mutations, 15.6% KMT2D mutations, 12.8% ARID1A mutations, 12.8% KEAP1 mutations, and 61.5% with DDR gene mutations (Table 2).

### 3.5. Therapy Response for Patients with TP53wt and TP53mut

**Figure 4:**
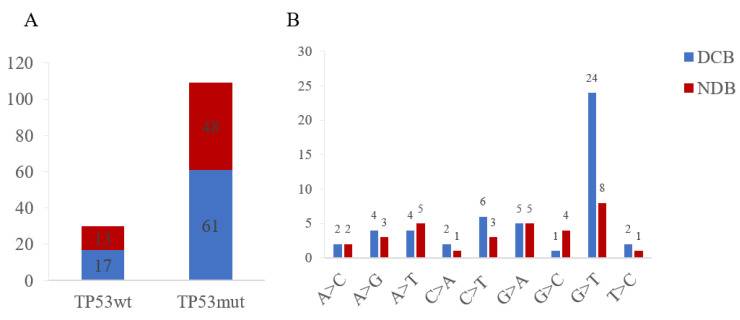
Clinical benefit (**A**) and nucleotide variation (**B**) of TP53wt and TP53mut cohorts. DCB, durable clinical benefit; NDB, no durable benefit.

NSCLC with TP53 mutations had a 56.0% rate of DCB, which revealed no significant difference with the wild-type cohort (Figure 4). Out of 17 patients in the TP53wt group, 3 who underwent immunotherapy were diagnosed with an adverse immune-related disease, compared with 9 out of 73 patients in the mutTP53 cohort. Significantly, patients with EGFR mutation had a lower DCB rate compared with the TP53 mutation cohort (*p* < 0.01). Furthermore, for TP53 missense mutation, nucleotide variation in G>T was achieved at a relatively higher DCB rate.

## 4. Discussion

Emerging evidence describes the regulatory mechanism of p53 in the metabolism balance and inflammatory response. It is recognized that LSCC frequently carries TP53 mutations related to carcinogen exposure and most p53 mutants are post-translationally modified at the same residues as wild-type status [11,12]. Hence, we summarized the demographics and characteristics of the censored 139 patients with advanced-stage lung cancer and found that more than half of the patients were of male sex and had a smoking history. Even though there was no obvious difference between TP53wt and TP53mut status in baseline characteristics, the TP53 mutant cohort revealed a significant association with male sex, adenocarcinoma differentiation, and smoking history.

TMB represents the overall number of mutations per megabase of DNA and is consequentially related to the number of neoepitopes that subsequently trigger T cell response. PD-L1 is a transmembrane cell surface protein, which is expressed on the tumor cells and inhibits T cell immune response [13]. Accordingly, TMB has been shown to be a potential predictor of response to immunotherapy across various tumor types, and increased TMB levels correlate with several factors, such as tobacco exposure and defective tumor suppressor gene TP53 [14]. In addition to PD-L1 expression level, our study revealed that the TP53mut cohort was much more obviously associated with TMB level, which may be related to lung cancer type and DNA replication errors mediation [15]. Furthermore, we found an inconsistency in the association between PD-L1 expression and TMB level, which is not unexpected (Figure S1).

Here, the majority of TP53 are missense mutations with amino acid changes mainly in the DNA binding domain (residues 102-292). The missense-mutant TP53 acquires gain-of-function (GOF) activities and can be broadly classified as DNA contact mutants and structural mutants, subsequently inactivating other tumor suppressive proteins and promoting tumor progression. Different from traditional hotspot mutation points [9], we identified that mutations in TP53 were scattered and the most frequent hotspots being codons 273(7/125), 158(6/125), and 175(4/125) in advanced lung cancer. Considerable evidence has solidified that the oncogenic function of mutp53 is not only caused by altered structure and properties of GOF, but also by losing its tumor suppressive activity. Thus, a smaller but considerable fraction of TP53 mutations is truncating mutations that impair the wild-type effect and complete loss-of-function (LOF) during tumor development [16].

Although mutations in the p53 gene exist in around half of all human cancers, detailed co-mutation analysis in the real world of advanced lung cancer is limited. Traditionally, p53 pathway activation controls the cellular response to different stress stimuli and determines cell fate under a specific context. Amplification of Mdm2/4 appears to promote tumor cell survival, drug resistance, invasion, and metastasis via E3 ubiquitin-mediated proteasome degradation. Ataxia telangiectasia mutated (ATM), ataxia telangiectasia, and Rad3-related protein (ATR) and the checkpoint kinases CHK1 and CHK2 stabilize p53 by disrupting p53-MDM2 interaction [17]. Numerous mutated genes correlated with tumor therapy response, such as EGFR, KRAS, ARID1A, and SMARCA4. Interestingly, we outlined the co-mutational status of these beneficial and noxious genes with TP53 in lung cancer and found a significantly different status of co-mutated DNA damage repair genes was detected in the TP53mut cohort, which may be related to better immune checkpoint inhibitor response and prognosis.

All patients with TP53wt and TP53mut showed no difference in durable clinical benefit, which may be due to limited samples and the short follow-up period of the analyzed data. Furthermore, a minimal degree of informative censoring was ruled out, leading to an underestimation of survival times. We revealed that 3 out of 17 patients in the TP53wt group were diagnosed with an adverse immune-related disease, compared with 9 out of 73 patients in the mutTP53 cohort who underwent immunotherapy. Thus, the relationship between adverse immune-related disease and mutated TP53 still needs further exploration. Furthermore, nucleotide variation in G>T achieved a relatively higher DCB rate, which might be related to oxidative stress and recognized as a predictor for TP53mut patients [18]. Nowadays, increased recognition of p53 has paved the way for various therapies targeting TP53 to restore normal p53 tumor suppressive function [19]. Studies describing artificial compounds that inactivated TP53 mutations provided a conceptual basis for the feasibility of mutant p53 reactivation [20].

## 5. Conclusions

In conclusion, this real-world retrospective study provides unparalleled statistical power to the properties of TP53wt and TP53mut in advanced-stage NSCLC. TP53 mutant was not only associated with male sex, adenocarcinoma differentiation, and smoking history, but also affected PD-L1 expression, TMB level, and co-mutant alterations. Our data also identified the prognostic value of nucleotide variation in G>T and molecular stratification on the basis of TP53 mutations should be broadened for NSCLC to optimize and modify clinical therapy choices.

## Figures and Tables

**Table 1:** Demographics and characteristics for patients with TP53wt and TP53mut in advanced lung cancer.

Variants	Total No.139	Patients with TP53wt, No. (%)	Patients with TP53mut, No. (%)	*p* Value (Chi-Square)	Patients with TP53“Other Mutations,” No. (%)	Patients with TP53 Missense Mutations, No. (%)	*p* Value (Chi-Square)
Age at study entry (y)				0.838			0.999
≤65	58(41.7)	13(43.3)	45(41.3)		17(41.5)	28(41.2)	
>65	81(58.3)	17(56.7)	64(58.7)		24(58.5)	40(58.8)	
Sex				*p* < 0.001			0.803
Male	102(73.4)	14(46.7)	88(80.7)		34(82.9)	54(79.4)	
Female	37(26.6)	16(53.3)	21(19.3)		7(17.1)	14(20.6)	
Histological type							
LUAD	83(59.7)	25(83.4)	58(53.2)	0.011	21(51.2)	37(54.4)	0.429
LSCC	43(30.9)	4(13.3)	39(35.8)		13(31.7)	26(38.2)	
Sarcomatoid carcinoma	13(9.4)	1(3.3)	12(11.0)		7(17.1)	5(7.4)	
Smoking history				0.003			0.629
No	38(27.3)	15(50)	23(21.1)		10(24.4)	13(19.1)	
Yes	101(72.7)	15(50)	86(78.9)		31(75.6)	55(80.9)	
PD-L1				0.015			0.603
<1%	20(14.4)	9(30)	11(10.1)		4(9.8)	7(10.3)	
1–49%	68(48.9)	14(46.7)	54(49.5)		18(43.9)	36(52.9)	
≥50%	51(36.7)	7(23.3)	44(40.4)		19(46.3)	25(36.8)	
TMB				*p* < 0.001			0.417
<10	95(68.3)	28(93.3)	67(61.5)		23(56.1)	44(64.7)	
≥10	44(31.7)	2(6.7)	42(38.5)		18(43.9)	24(35.3)	

TP53mut, TP53 mutated; TP53wt, TP53 wild-type; LUAD, lung adenocarcinoma; LSCC, lung squamous cell carcinoma; TMB, tumor mutational burden; PD-L1, programmed death receptor ligand 1 (PD-L1).

**Table 2:** Co-mutational status for patients with TP53wt and TP53mut in advanced lung cancer.

Variants	Total No. 139	Patients with TP53wt, No. (%)	Patients with TP53mut, No. (%)	*p* Value (Chi-Square)	Patients with TP53 Missense Mutations, No. (%)	Patients with TP53 “Other Mutations,” No. (%)	*p* Value (Chi-Square)
Beneficial genes							
PBRM1							
Wild type	130(93.5)	26(86.7)	104(95.4)	0.085	66(97.1)	38(92.7)	0.004
Mutant	9(6.5)	4(13.3)	5(4.6)		2(2.9)	3(7.3)	
KRAS							
Wild type	115(82.7)	23(76.7)	92(84.4)	0.321	59(86.8)	33(80.5)	0.382
Mutant	24(17.3)	7(23.3)	17(15.6)		9(13.2)	8(19.5)	
ARID1A							
Wild type	122(87.8)	27(90.0)	95(87.2)	0.674	61(89.7)	34(82.9)	0.306
Mutant	17(12.2)	3(10.0)	14(12.8)		7(10.3)	7(17.1)	
SMARCA4							
Wild type	126(90.6)	27(90.0)	99(90.8)	0.891	63(92.6)	36(87.8)	0.396
Mutant	13(9.4)	3(10.0)	10(9.2)		5(7.4)	5(12.2)	
DDR pathway							
Wild type	54(38.8)	12(40.0)	42(38.5)	0.884	18(26.5)	24(58.5)	*p* < 0.001
Mutant	85(61.2)	18(60.0)	67(61.5)		50(73.5)	17(41.5)	
Noxious genes							
EGFR				0.061			0.849
Wild type	102(73.4)	18(60.0)	84(77.1)		52(76.5)	32(78.0)	
Mutant	37(26.6)	12(40.0)	25(22.9)		16(23.5)	9(22.0)	
KEAP1							
Wild type	124(89.2)	29(96.7)	95(87.2)	0.137	59(86.8)	36(87.8)	0.875
Mutant	15(10.8)	1(3.3)	14(12.8)		9(13.2)	5(12.2)	
MDM2/MDM4							
Amplification	7(5.0)	3(10.0)	4(3.7)	0.161	2(2.9)	2(4.9)	0.602
No amplification	132(95.0)	27(90.0)	105(96.3)		66(97.1)	39(95.1)	
11q13							
Amplification	8(5.8)	1(3.3)	7(6.4)	0.521	4(58.8)	3(7.3)	0.767
No amplification	131(94.2)	29(96.7)	102(93.6)		64(94.1)	38(92.7)	
JAK1/JAK2							
Wild type	126(90.6)	29(96.7)	97(89.0)	0.201	62(91.2)	35(85.4)	0.348
Mutant	13(9.4)	1(3.3)	12(11.0)		6(8.8)	6(14.6)	
CTNNB1							
Wild type	133(95.7)	27(90.0)	106(97.2)	0.084	66(97.1)	40(97.6)	0.877
Mutant	6(4.3)	3(10.0)	3(2.8)		2(2.9)	1(2.4)	
STK11							
Wild type	127(91.4)	28(93.3)	99(90.8)	0.665	61(89.7)	38(92.7)	0.602
Mutant	12(8.6)	2(6.7)	10(9.2)		7(10.3)	3(7.3)	
KMT2D							
Wild type	120(86.3)	28(93.3)	92(84.4)	0.207	58(85.3)	34(82.9)	0.741
Mutant	19(13.7)	2(6.7)	17(15.6)		10(14.7)	7(17.1)	
DNMT3A							
Wild type	133(95.7)	29(96.7)	104(95.4)	0.765	65(95.6)	39(95.1)	0.911
Mutant	6(4.3)	1(3.3)	5(4.6)		3(4.4)	2(4.9)	
PTEN							
Wild type	127(91.4)	28(93.3)	99(90.8)	0.665	63(92.6)	36(87.8)	0.396
Mutant	12(8.6)	2(6.7)	10(9.2)		5(7.4)	5(12.2)	

Chromosome 11q13 amplification panel: CCND1, FGF3, FGF4, and FGF19. DDR gene panel: MLH1, MSH2, MSH6, PMS1, PMS2 (MMR); ERCC2, ERCC3, ERCC4, ERCC5 (NER); BRCA1, MER11A, NBN, RAD50, RAD51, RAD51B, RAD51D, RAD52, RAD54L (HR); BRCA2, BRIP1, FANCA, FANCC, PALB2, RAD51C, BLM (FA); ATM, ATR, CHEK1, CHEK2, MDC1 (Checkpoint); POLE, MUTHY, PARR1, RECQL4, POLD1 (Others).

## Data Availability

All data, models, and code generated or used during the study appear in the submitted article.

## Supplementary Materials

The following supporting information can be downloaded at: https://www.mdpi.com/article/10.3390/curroncol29100582/s1, Figure S1: The association between TMB and PD-L1 levels in TP53mut cohort.
