# Supplementary figures and images for: TP53 Mutation Mapping in Advanced Non-Small Cell Lung Cancer: A Real-World Retrospective Cohort Study
Source: Curr Oncol. 2022 Oct 4;29(10):7411–9. doi: 10.3390/curroncol29100582 (PMC9599964; doi:10.3390/curroncol29100582)

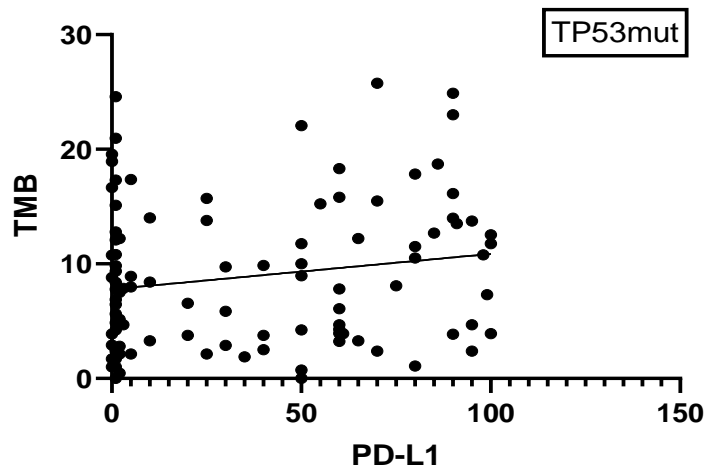

Supplement Figure S1. The association between TMB and PD-L1 levels in TP53mut cohort.

Supplement: Supplementary file 1 [file curroncol-29-00582-s001.zip › curroncol-1919596-supplementary.pdf]
